# Local Anæsthesia

**Published:** 1886-06

**Authors:** 


**Local Anæsthesia.**—Dr. Govan stated, at the N. Y. State Medical Association, that he had used aniline oil for the purpose of producing local anæsthesia when laying open felons and performing other minor operations. There was absolutely no pain, even in cutting down to the bone, when the finger had been dipped for a short time in the oil.

